# Two Faces of Vitamin C—Antioxidative and Pro-Oxidative Agent

**DOI:** 10.3390/nu12051501

**Published:** 2020-05-21

**Authors:** Julia Kaźmierczak-Barańska, Karolina Boguszewska, Angelika Adamus-Grabicka, Bolesław T. Karwowski

**Affiliations:** DNA Damage Laboratory of Food Science Department, Faculty of Pharmacy, Medical University of Lodz, ul. Muszynskiego 1, 90-151 Lodz, Poland; julia.kazmierczak-baranska@umed.lodz.pl (J.K.-B.); karolina.boguszewska@umed.lodz.pl (K.B.); angelika.adamus@umed.lodz.pl (A.A.-G.)

**Keywords:** vitamin C, antioxidants, pro-oxidative factor, anticancer activity, DNA lesions, radioprotective properties

## Abstract

Vitamin C has been known for decades. It is common in everyday use as an element of the diet, supplementation, and a preservative. For years, research has been conducted to precisely determine the mechanism of action of ascorbate in the cell. Available results indicate its multi-directional cellular effects. Vitamin C, which belongs to antioxidants scavenging free radicals, also has a ‘second face’—as a pro-oxidative factor. However, whether is the latter nature a defect harmful to the cell, or whether a virtue that is a source of benefit? In this review, we discuss the effects of vitamin C treatment in cancer prevention and the role of ascorbate in maintaining redox balance in the central nervous system (CNS). Finally, we discuss the effect of vitamin C supplementation on biomarkers of oxidative DNA damage and review the evidence that vitamin C has radioprotective properties.

## 1. Introduction

Scurvy is one of the earliest described diseases. It wreaked havoc on sailors in the 16–18th century until the remedy proposed by surgeon James Lind was to be lemon juice [1]. However, it was not until the first half of the 20th century that ascorbic acid (AA) was extracted for the first time from pepper and adrenal gland extracts by the Hungarian biochemist Albert Szent-Gorgyi in 1928 (hexuronic acid), for which he was awarded the Nobel Prize (1937) in Physiology or Medicine. At that time, knowledge about vitamin C (VC) has expanded tremendously. Ascorbic acid is one of the most frequently supplemented vitamins. The food industries use the stabilizing and antioxidative properties of this compound [2]. Much is known about the positive impact of its action on the human body, but the dark pro-oxidative side of the power of vitamin C is also known. In this review, the authors focus on vitamin C in the context of not only antioxidant properties but also its pro-oxidative potential. The first part presents the role of ascorbate in cancer metabolism and biology. The authors emphasize that vitamin C alone does not have a therapeutic effect on cancer itself; however, it is beneficial as a supplement for patients undergoing chemotherapy and radiation therapy. It then describes the role of ascorbate in the nervous system, where, acting as the main antioxidant, it can support potential therapies by reducing the level of reactive oxygen species (ROS). The last part of the review focuses on oxidative DNA damage, the role of ascorbate in this process, and its effect on DNA repair proteins.

## 2. Vitamin C: Significant Element of Cellular Metabolism

**Figure 1:**
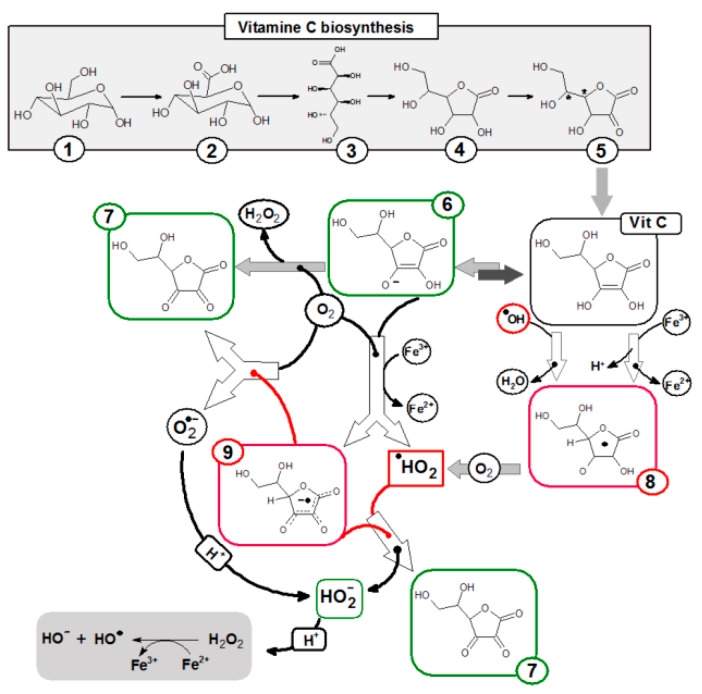
Pathway of ascorbic acid synthesis in animals (except humans, primates, and guinea pigs) and chemical transformation pathway of ascorbic acid. (**1**) α-d-glucopyranose; (**2**) d-glucuronic acid; (**3**) Gulonic acid; (**4**) l-gulonolactone; (**5**) 2-keto-l-gulonolactone; (**6**) Ascorbate anion; (**7**) Dehydroascorbic acid; (**8**) Ascorbyl radical; (**9**) Ascorbyl radical anion.

The human body as one of the few must provide l-ascorbic acid in the diet, this is associated with the lack of the enzyme l-gulonolactone oxidase, which catalyzes the last stage of ascorbic acid biosynthesis by oxidation of l-gulonolactone. Most mammals synthesize from d-glucose, in the glucuronic acid pathway (Figure 1). In plants, vitamin C production is more complex, there are two main pathways of vitamin C biosynthesis from d-glucose or from d-galactose [3].

The recommended daily dose of vitamin C is on average 75 mg for women and 90 mg for men [4]—a proper diet provides it, which is why vitamin C deficiency is rare nowadays and applies to special cases such as malnutrition, malabsorption, kidney disease, and smokers. However, there are suggestions that recommended doses are too low, it is associated with the current lifestyle, stress, and an incorrect diet rich in highly processed products and sugar, which inhibits the absorption of ascorbic acid. Reduced absorption of dehydroascorbic acid (DHA) at high glucose concentration in the culture medium due to competition of glucose and ascorbate for GLUT-1 transporters was observed in some types of cells (e.g., muscle cells) [5]. The concentration of vitamin C in plasma depends on the supply, health, absorption, and excretion, reaching at ~50 μM range and can reach ~150 μM. Intracellular vitamin C levels can reach significantly higher concentrations, e.g., in lymphocytes of about 4 mM [6] or in neurons of up to 10 mM [7]. The highest concentrations are achieved in the adrenal glands and pituitary gland [7,8]. Interestingly, the bioavailability of ascorbic acid is dose-dependent (higher absorption is observed at a lower dose) and is actively transported through cell membranes. Active vitamin C transporters (SVCT1 and SVCT2) are known that can modify the amount of VC serum in relation to dietary supply [9]. SVCT1 isoform for very high transportability, enabling massive uptake of vitamin C. SVCT1 expression primarily concerns epithelial cells of the digestive system. In turn, the SVCT2 isoform is expressed in highly specialized cells such as neurons or placenta and is characterized by high affinity at low transport efficiency [10]. SVCT2 seems to be particularly important because point mutations in this protein gene (*SLC23A2*) lead to a decrease in ascorbate concentration in plasma [11]. *SLC23A2* mutations have been observed to be associated with an increased risk of the acute coronary syndrome, gastric cancer, hematologic malignancies, and glaucoma [12]. In addition, transport of the oxidized form—DHA to the cell occurs through glucose transporters (GLUT), which in the cell is again reduced to vitamin C [13].

The basic biological function of ascorbic acid is, in addition to the cofactor function of several enzymes (e.g., dopamine B-monooxygenase or prolyl 4-hydroxylase and lysyl hydroxylase [14]), to protect cell components against free radicals which are commonly formed during the metabolism. Ascorbate is one of the hydrophilic antioxidants that accumulates in the aqueous phase of the cell. The hydroxyl groups at the double bond in the lactone ring are donors of protons and electrons, turning into the diketone moiety of DHA (Figure 1), which determines the strong reducing properties of ascorbic acid and protects other cellular components from oxidation. These hydroxyl groups are reactive, among others towards singlet oxygen, hydrogen peroxide, peroxide radicals, and hydroxyl radicals. One-electron oxidation of ascorbic acid leads to the formation of a stable and non-reactive ascorbic radical (A^●^). Loss of another electron by the ascorbic radical leads to its transformation into dehydroascorbic acid, which has the same biological activity as the reduced form (Figure 1). Hydrolysis of dehydroascorbic acid to 2,3-diketogulonic acid is irreversible and leads to loss of antioxidant properties and its degradation. However, DHA can be reduced back to ascorbic acid. This reduction is carried out by various enzymes, for example dehydroascorbate reductase, an enzyme that uses reduced glutathione as a co-substrate, while glutathione modulates the redox properties of vitamin C [15,16,17,18]. The reduction of l-ascorbic acid occurs under the influence of hydrogen sulfide or hydrogen iodide effects.

**Figure 2:**
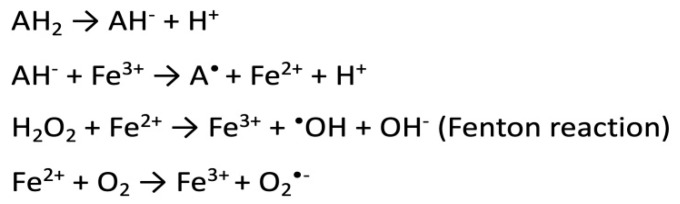
Scheme of hydroxyl radical generation according to Fenton’s predictions. AH_2_—ascorbic acid; AH^−^—ascorbate anion; A^•^—ascorbyl radical; ^•^OH—hydroxyl radical; O^−^_2_—superoxide anion.

The pro-oxidative activity of ascorbic acid (Figure 2) is associated with the interaction with transition metal ions (especially iron and copper). Under conditions of high, millimolar ascorbate concentration, vitamin C catalyzes the reduction of free transition metal ions, which causes the formation of oxygen radicals. Reduced iron ions react with hydrogen peroxide to form reactive hydroxyl radicals or peroxide ions. This reaction occurs in the presence of oxygen [14,19]. In addition, an excess of vitamin C of 3 mg/day can impair the absorption of iron, copper or vitamin B12 [8]. While there is extensive evidence of vitamin C pro-oxidative abilities in the presence of transition metals in vitro, there is no convincing and unambiguous evidence of such in vivo activity. This may be the result of tightly controlled metabolism of metals such as Fe or Cu, which are sequestered by specific proteins [20].

## 3. Vitamin C: Anti-Cancer Potential

The most important property underlying the biological activity of ascorbic acid is its reversible oxidation, and reduction capacity described in the previous chapter. Ascorbate, by reducing metal ions such as iron, or copper, ensures their catalytic activity. The reduction of Fe^3+^ to Fe^2+^ iron enables the implementation of various enzymatic processes dependent on iron ions especially those that play an important role in DNA synthesis or epigenetics. One of them is a post-translational modification of collagen, proline, and lysine hydroxylase, in the active center they contain Fe^2+^ ion, which is why vitamin C deficiency leads to disruption of connective tissue function, especially the walls of blood vessels.

**Figure 3:**
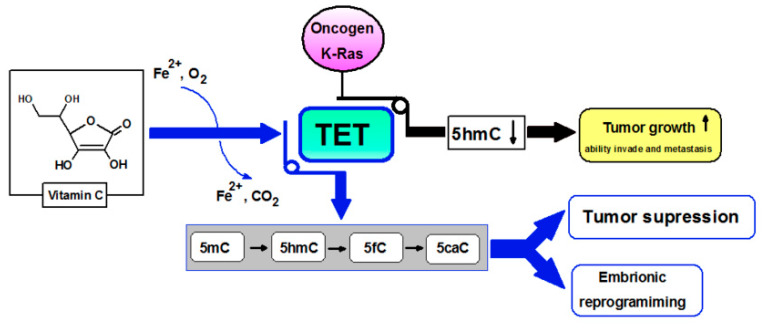
Vitamin C ensuring the proper functioning of TET proteins according to [24,27,28,29]. K-Ras, oncogene product of *KRAS* gene; TET, methylcytosine oxidase ten-eleven translocation proteins; 5-mC, 5-methylcytosine; 5-hmC, 5-hydroxymethylcytosine; 5-fC, 5-formylocytosine; 5-caC, 5-carboxycytosine.

Another is the post-translational regulation of the level of HIF1 transcription factor (hypoxia-inducible factor 1) by enzymes from the group of hydroxylases: Fe^2+^/2-oxoglutarate (2OG)-dioxygenase-dependent requiring ascorbate for action. The lack of necessary cofactors, i.e., ascorbate, or iron, interferes with the activity of hydroxylases, leading to increased stabilization and activation of HIF1 [21]. HIF1 regulates the transcription of hundreds of genes coding for proteins involved in various aspects of cancer biology, e.g.: cell immortality, angiogenesis, or resistance to chemotherapy, and radiation therapy. The consequence of the high rate of proliferation is impaired access of cancer cells to nutrients, including glucose, and oxygen, which causes a change in cellular metabolism to anaerobic [22]. The level of HIF1 in cells depends on the amount of oxygen, it can increase as a result of activation of oncogenes, it is also regulated by the availability of ascorbate which modulates the activity of hydroxylases. Vitamin C is considered one of the epigenetic modulators (Figure 3) because it is a cofactor of enzymes involved in the reprogramming of hydroxylase cells, e.g., Fe^2+^/2-oxoglutarate (2OG)-dioxygenase [21] or TET proteins (methylcytosine oxidase ten-eleven translocation proteins) [23,24]. Research on embryonic stem cells (ES) shows the addition of VC to the culture, induces DNA demethylation in a TET1/TET2-dependent manner, and expression of key genes for germline development [25]. The authors showed that ascorbate deficiency reduces the expression of these genes. The lack of ascorbate seems to have not affected the general development of gonads but reduces the number of germ cells and results in abnormal expression of TET1-dependent germ cell genes. Vitamin C-deficient germ cells have a different gene expression profile compared to controls. Two-thirds of DMR (differentially methylated regions) undergo hypermethylation in the case of germ cells forming in the absence of ascorbate [25]. The authors conclude that vitamin C is required for proper transcription program coordinated by TET1 and is necessary for normal fertility. It is believed that vitamin C affects the activity of TET proteins not only by enabling the reduction of Fe^3 +^ to Fe^2+^ but also as a cofactor for the correct folding of the enzyme [26]. TET genes play the role of suppressors [27], it was observed that in many cancers *TET* genes mutate and their expression is reduced [28]. It was shown that TET expression can be inhibited by the K-Ras oncogene, leading to a reduction of 5-hydroxymethylcytosine (5-hmC) levels in favor of 5-methylcytosine (5-mC). As a result, this leads to a decrease in the expression of proapoptotic genes, e.g., DAPK (death-associated protein kinase) [29]. TET proteins play an important role in DNA demethylation, catalyzing the conversion of 5-mC to 5-hmC (Figure 3). The pattern of DNA methylation depends on the type of tissue, and the stage of development of the body. A strong reduction of 5-hmC in different types of cancers was observed [30], however, it is not always associated with TET mutations. The loss of 5-hmC may also result from, e.g., mutation in the Wilms tumor suppressor gene (WT1 suppressor gene 1) [31] or the intensive rate of tumor cell proliferation, which leads to a depletion of 5-hmC [32]. It was observed that a decrease in ascorbate concentration with normal TET gene expression has a significant effect on a decrease in 5-hmC concentration in the lungs, liver, and brain, but probably also in other tissues [26]. However, with reduced TET1/2 gene activity, the presence of ascorbate does not affect the level of 5-mC.

Epidemiological studies indicate a relationship between serum vitamin C levels, and the risk of diseases such as cancer, and cardiovascular disease. A higher mortality rate (for various reasons) was observed among patients with vitamin C deficiency compared to the control group. [33]. There are data indicating the higher the concentration of vitamin C in the serum tested, the lower the risk of cardiovascular disease [34]. A similar correlation is observed in the case of stroke risk. People with low ascorbate are at twice the risk of stroke compared to people with normal ascorbate in their serum [35]. No strong evidence for a correlation between VC levels and cancer risk. Decreased ascorbate levels are observed in cancer patients but there is no explanation as to whether this is the cause or effect of the disease. No positive effect of additional vitamin C supplementation on the prevention and inhibition of cardiovascular disease or reduction of incidence and mortality due to cancer was observed [12,36].

Many studies indicate the toxicity of ascorbate to cancer cells, although the exact mechanism of this effect has not been elucidated. Much evidence indicates that the underlying phenomenon is the pro-oxidative activity of ascorbate, which induces the formation of H_2_O_2_ and oxidative stress [37,38,39]. The pro-oxidative activity of ascorbate depends on the presence of iron ions and, interestingly, ROS promote the increase of labile iron pool (LIP) levels [40]. Furthermore, elevated iron levels recognized in cancer cells activate iron-dependent proteins that enable adaptation to hypoxia and stimulate cell proliferation. In normal cells, properly functioning free radical neutralization systems, the limited availability of LIP means that the antioxidant face of vitamin C prevails [41]. Interest in high doses of vitamin C in the treatment of cancer has its source in vitro studies which have shown that different tumor cell lines (A431—epidermoid carcinoma; Panc-1—pancreatic; HeLa—cervical; HT29—colonic; and MCF7—breast carcinoma cells) are sensitive to increased doses of vitamin C [42]. In a 2012 study, Chen et al. showed that five of the six tested prostate cancer lines are sensitive to millimolar ascorbate levels. The authors explain the cytotoxicity of high concentrations of ascorbate towards cancer cells by the formation of H_2_O_2_ in the extracellular space. This leads to oxidative stress-induced autophagy of cancer cells [43]. There is evidence that ascorbate can induce cancer cell death. Apoptosis is a process that is dependent on caspase but also independent. It is known that the mitochondrion in response to proapoptotic signals releases apoptosis inducing factor AIF, the effector of cell death. AIF is directed to the cell nucleus where it induces chromatin condensation and DNA fragmentation. Hong et al. showed that ascorbate induced cell death by AIF in breast cancer cells [44]. Similar observations apply to melanoma cells, ascorbate at a physiological concentration (100 µM) induced apoptosis by inhibiting clusterin expression (CLU). Overexpression of clusterin protects melanoma cells from apoptosis [45]. Interesting data indicate the possibility of inducing cancer cell death by autoschizis by a combination of vitamin C and vitamin K. The peroxides generated by vitamin C lead to peroxidation of plasmalemma or organelle membranes e.g., mitochondria, which results in the undergoes an autoschizis. The autoschizis is a type of necrosis, characterized by excessive membrane damage, damage to organelles, and an intact nucleus with decondensed chromatin. As a consequence, the cell shrinks, and dies inducing local inflammation [46,47].

Support the action of vitamin C in cisplatin therapy has been reported. Cisplatin (CisPt) is one of the first anti-cancer drugs used to treat epithelial cancers [48]. The cytotoxicity of platinum complexes is due to covalent binding to DNA and the formation of intra- and inter-stranded cross-links, which consequently leads to inhibition of DNA synthesis and replication and eventually to apoptosis of cancer cells. However, the use of cisplatin is burdened with numerous adverse side effects (neurotoxicity, nephrotoxicity, hepatotoxicity) [49], and more often, secondary drug resistance. The results obtained for cervical cancer cells show that the combination of CisPt treatment with vitamin C, enhances the therapeutic effect of cisplatin, and has a strong antiproliferative effect [50]. Tumors show a decrease in the activity of various antioxidant enzymes, especially catalase (CAT), H_2_O_2_ decomposing enzyme, which makes them particularly sensitive for hydrogen peroxide and other ROS [51]. Vitamin C introduced from CisPt has a pro-oxidative effect, generates H_2_O_2_ which enhances oxidative stress, and the genotoxic effect of CisPt on DNA. Furthermore, the authors observed that the administration of vitamin C with CisPt leads to increased p53 expression in cervical cancer cells and makes them more susceptible to apoptosis [50]. The main tumor suppressor—p53 protein—is a transcription factor that plays a major role in initiating the cell’s response to stress caused by DNA damage, hypoxia, and abnormal proliferative signals. P53 decides whether the cell will be arrested in the cell cycle and start repairing damaged DNA, or start apoptosis and it depends on the severity of stress and the extent of the damage. Previously, anti-tumor ascorbate activity was shown by stabilizing the p53 protein. The addition of a pharmacological concentration of ascorbate to *p53^+/+^* HTC116 cells (colon cancer) caused an extension of the p53 half-life by induction of MDM2 degradation. Furthermore, the authors suggested that the tumor response to ascorbate treatment may depend on p53 expression in the cell line under study and the efficacy may be enhanced by combining vitamin C therapy with another cancer drug [52]. In view of this, it appears that the combination of VC and CDDP can be very useful in increasing therapeutic efficacy against the cervical cancer cell line and suggests the benefits of vitamin C.

Colon carcinoma (CRC) with *KRAS* or *BRFA* mutations appears to be another example of a high dose vitamin C sensitive tumor [53]. Research results carried out on cell models suggest that *KRAS* or *BRAF* mutant CRC cells showing increased expression of GLUT glucose transporters are characterized by increased uptake of DHA into cells and this leads to a reduction in cancer cell viability. Cancer cells have a different metabolism. Due to the developing oxygen deficiency, the metabolism is shifted towards anaerobic glycolysis. The high energy requirements of the cancer lead to an intensification of glycolysis as well as an increase in glucose uptake using GLUT transporters, whose increased expression in cancer cells is observed. As mentioned above, GLUTs are the path through which DHA enters the cell. The authors showed that by hyperaccumulation of DHA, via GLUT-1, ascorbate causes severe oxidative stress, and causes cell death [53]. Furthermore, GLUT overexpressing cancer cells are sensitized to ascorbate. However, it remains unclear whether accumulation of DHA, or modulation of enzyme activity, or the pro-oxidative properties of ascorbate leading to the formation of H_2_O_2_ determines this [54].

The potential for vitamin C anti-cancer properties has been fortified by a series of studies conducted on animal models. It was observed that oral vitamin C supplementation only achieves the physiological concentration of ascorbate in the blood, while parenteral administration allows higher pharmacological concentrations of about 20 mM in the blood [55]. Intraperitoneal injection of high concentrations of vitamin C (1 g/kg) inhibited hepatoma growth in mice. The authors conclude that high levels of vitamin C generate H_2_O_2_ induces tumor cell necrosis. Reduced ability to metabolize H_2_O_2_ by cancer cells compared to normal cells was demonstrated [56]. Nuclear and mitochondrial DNA is the main goal of vitamin C’s pro-oxidative action. However, less effective mitochondrial repair systems increase their sensitivity. Mitochondrial dysfunction can induce cell death, and this is extremely beneficial in the ‘fight’ against cancer. Normal cells have an efficient apparatus for antioxidant enzymes, while cancer cells have reduced levels. Studies conducted on 10 types of normal cell lines and 15 cancer lines support the thesis that reduced catalase activity increases the sensitivity of cancer cells, and catalase can be a marker of the effectiveness of vitamin C therapy. Other animal studies have also confirmed the effectiveness of vitamin C in inhibiting the growth of tumors such as glioblastoma [57], pancreatic [58], cervical [59], and colorectal cancer [53]. Studies have shown impaired tumor progression after via intraperitoneal injection of high-dose vitamin C (4 g/kg) without adversely affecting normal tissues.

**Figure 4:**
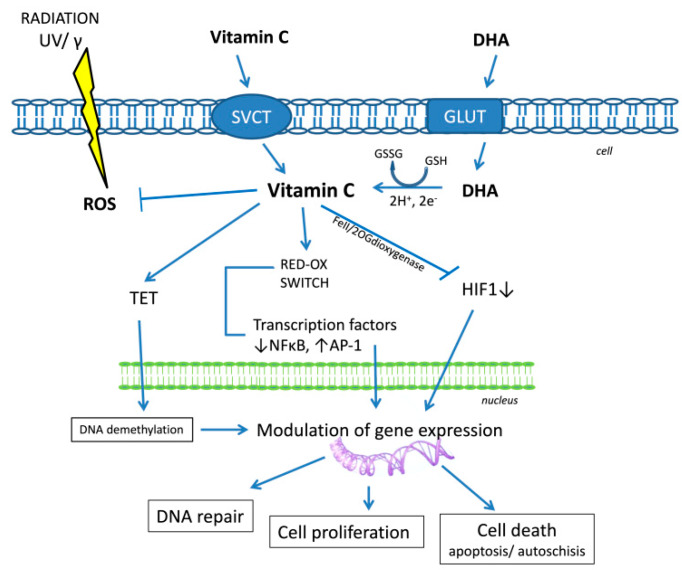
Biological function of vitamin C according to [15,21,23,44,47]. SVCT, sodium-dependent vitamin C transporter; DHA, dehydroascorbic acid; GLUT, Glucose transporter; GSH, glutathione; GSSG, glutathione disulfide; HIF-1, Hypoxia-inducible factor 1; TET, methylcytosine oxidase ten-eleven translocation proteins; NF-κB, nuclear factor kappa-light-chain-enhancer of activated B cells; AP-1, activator protein 1.

Despite promising observations in vitro and in vivo that vitamin C has anti-cancer potential, there is no clear evidence of clinical efficacy. Vitamin C has been found to support chemo/radiotherapy, improving treatment effects and patients’ quality of life by reducing adverse side effects [60,61]. Other clinical studies have not shown significant influence on various cancers: breast [62], colorectal, pancreatic, biliary tract, gastric [63]. However, other results of clinical studies show that the administration of antioxidants during cancer therapy caused a decrease in the level of markers of oxidative stress in blood serum [64]. The general disease state of cancer patients, dietary deficiencies, the body’s greater antioxidants requirements due to increased oxidative stress cause depletion of antioxidants such as vitamin C. Intravenous infusions of vitamin C are proposed to supplement deficiencies and a factor supporting the eradication of, e.g., CSC cancer stem cells [65]. This appears to be of particular importance in the case of therapies for cancers resistant to e.g., doxycycline [66]. CSC is a small population of cancer cells considered to be the primary tumor source for recurrence. These cells are characterized by low proliferative potential and elastic metabolism which, when using therapies directed at fast-dividing cells, means that they are often resistant to the standard treatment regimen [65,67]. Despite the lack of clear evidence of the therapeutic, anti-cancer effect of vitamin C, it seems that it can support this unequal fight, and improve the quality of life of patients undergoing chemo/radiotherapy. These reports explain the ongoing interest in vitamin C in the fight against cancer. However, the primary element should be adequate prevention, and nutrition education (Figure 4).

## 4. Vitamin C: Main Antioxidant of the CNS

The presence of ascorbate is extremely important for homeostasis and the proper functioning of the central nervous system (CNS). The brain consumes a large portion of glucose, about 25%, and about 20% oxygen, which implies rapid metabolism and increases free radical production. Reactive oxygen species (ROS) produced in physiological concentrations, safe for the cell, are involved in the processes of neuromodulation, neurotransmission and synapse plasticity control. The brain, being an organ intensively metabolizing oxygen and having relatively weak protective antioxidant mechanisms, is particularly susceptible to oxidative stress [68]. To maintain redox balance, the brain depends on high levels of antioxidants, and the most abundant antioxidant present in brain tissue is ascorbate (Table 1), in addition to glutathione. The main line of defense of neuron cells against ROS is neighboring astrocytes and glial cells. Neurons release ascorbate (dehydroascorbate) oxidized by ROS into the extracellular environment, captured by neighboring glial cells that convert it to reduced ascorbate taken back by neurons [7]. This recycling system allows the brain to maintain high levels of ascorbate. Ascorbate is mainly present in nerve cells, while glutathione in glial cells [69]. Aging or the development of neurodegenerative diseases is associated with increased levels of oxidative stress and redox imbalance resulting from deficiencies of antioxidants such as vitamin C. Ascorbate appears to be a significant neuroprotector [70]. It was observed that ascorbate inhibits the binding of the glutamate neurotransmitter to synaptic receptors, modulates the activity of glutamate receptors, reduces the stimulation of NMDA receptors, reduces the level of free radicals generated by the release of glutamate, and thus protects neurons from the so-called glutamate excitotoxicity. It is believed that high-dose vitamin C supplementation may be protective and reduce the size of ischemia [69,71].

Mitochondrial dysfunction, elevated oxidative stress, amyloid plaque formations are processes involved in the pathogenesis of Alzheimer’s disease [73]. Studies in mice (transgenic, lacking the *Gulo* gene) have shown that prolonged vitamin C supplementation significantly reduces the formation of amyloid plaque [74]. Importantly, ascorbate seems to be crucial for the proper functioning, and protection of mitochondria (so important for neurons). Mitochondria is a natural place for ROS, it is easy to damage mtDNA, which without protective histones is all the more sensitive to damage. Vitamin C appears to quench ROS, which contributes to the stabilization of the mitochondrial membrane [75]. A stable and integral mitochondrial membrane is a crucial element of cell homeostasis. It is known that a disorder in this area can lead to the release of cytochrome C, which is a proapoptotic signal for the cell. Last but not least, the enzyme for which the cofactor is ascorbate is dopamine β-hydroxylase (DBH), which catalyzes the formation of noradrenaline from dopamine. The key role of vitamin C in the synthesis of catecholamines indicates how important factor affecting the proper functioning of CNS is vitamin C [76].

Studies on neonatal vitamin C deficiency were conducted on guinea pigs. These animals are dependent on dietary intake of vitamin C, as are humans who cannot synthesize VC internally. The study describes that vitamin C is retained by the brain in the event of its general deficiency. Animals with vitamin C deprivation in their diet for 3 weeks show an ascorbate severe reduction in plasma and liver, up to 1% compared to the control group, while in brain cells up to 30%. The authors assume that ascorbate is to protect brain cells that are very susceptible to oxidative damage [77].

## 5. Vitamin C and Oxidative DNA Damage

The presence of free radicals in the cell is inextricably linked to its metabolism, they are an important element in accomplished the signaling or defense functions of the cell. Nevertheless, the lack of oxidative balance in the cell leads to the accumulation of free radicals, which in the absence of cellular antioxidants disrupt cellular functions, and lead to related disorders with aging, degeneration, or carcinogenicity.

**Figure 5:**
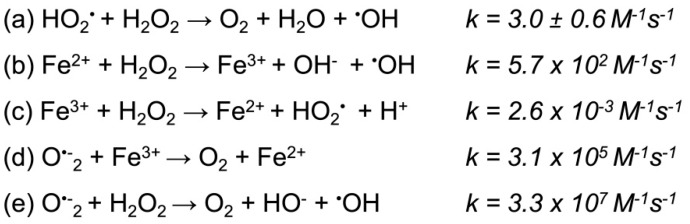
Haber–Weiss reaction scheme. The Haber–Weiss reaction generates hydroxyl radicals from hydrogen peroxide, and superoxide catalyzed by iron ions; *k*, reaction rate coefficient. (**a**) The reaction is too slow and cannot affect cellular processes. (**b**) A catalyst (iron) was used, which was already known in the Fenton reaction. (**c**) Iron ions formed in the Fenton reaction were reduced to iron ions by hydrogen peroxide. (**d**) In order for the reaction to proceed at a faster rate, a reducing agent was added: superoxide anion radical. (**e**) As a result of the b and d reactions and iron ions, the Haber–Weiss reaction was obtained, which proceeds at a high rate according to [78,79,80].

As mentioned above, iron is an essential catalytic element for the cell to function properly. Under physiological conditions, iron is extensively sequestered by iron binding proteins such enzymes, by iron transporting protein to cells—transferrin or by iron storage protein in cells—ferritin. This is the first line of defense against the formation of excessive amounts of free radicals (Figure 5, according to [78,79,80]).

Under pathological conditions, an uncontrolled release of iron may occur, as is the case with thalassemias. Iron overload is observed in this hemolytic anemia in [81]. Another disease, hemochromatosis, is a metabolic syndrome in which excessive storage of Fe in tissues occurs. The consequence is damage to cellular structures, and organs due to increased oxidative stress [82]. The pro-oxidative activity of vitamin C depends mainly on the availability of Fe. Iron reduced by ascorbate to Fe^2+^ easily reacts with oxygen, which in the Fenton reaction leads to the formation of reactive oxygen species and H_2_O_2_ which in reaction with Fe^2+^ generates a highly reactive hydroxyl radical [16]. Nevertheless, it is believed that in normal states H_2_O_2_ is efficiently and quickly utilized by the appropriate enzyme systems that fail in cancer cells. In tumor cells, inhibition of the activity of enzymes that neutralize oxidative stress e.g., catalase and superoxide dismutase, is observed [51], therefore the pro-oxidative potential of ascorbate is considered in the context of cells with impaired metabolism, e.g., cancer cells.

The possibility of ascorbate acting under strictly defined conditions as a pro-oxidant may give rise to doubts, is it really such an excellent antioxidant for DNA, and other cellular components? Does it effectively protect DNA? The presence of free radicals in the cell is a natural phenomenon associated with the implementation of aerobic metabolism. Both the decrease and excessive increase in ROS levels (including H_2_O_2_) in a normal cell is detrimental because they are an important signaling factor. Too low a level of ROS can inhibit proliferation or differentiation, while excessive amounts result in hyperproliferation [83]. The maintenance of redox balance is also extremely important for an unstable tumor cell: increased ROS levels needed for cancer progression, but excessive ROS levels can induce death [84]. Cancer cells are counteracted by upregulating antioxidant systems, for example enhance NADPH (reduced nicotinamide adenine dinucleotide phosphate) production to maintain antioxidant capacity. Therefore, induction of oxidative stress in cancers exhausts their antioxidant mechanisms and leads to apoptosis. Ascorbate can modulate the response to oxidative stress and DNA damage by altering redox signaling. DHA in the cell is oxidized while oxidizing glutathione (GSH) and generating H_2_O_2_. GSH regeneration proceeds in a manner dependent on NADPH. In this way, high doses of ascorbate depleting cellular GSH and NADPH lead to elevate ROS level in cancer cells, changes in redox signaling and death. In contrast, an increase in glutathione levels in peripheral blood lymphocytes after vitamin C supplementation was observed [85]. This indicates the beneficial and protective effect of vitamin C. Deficiency of GSH in lymphocytes increases the vulnerability to damage so important in the context of side effects of chemotherapy or radiation therapy. Moreover, the protective effect of vitamin C on the tissues of the heart, liver, and kidneys was demonstrated [86]. Studies on the chronic hHcy rat model have shown that VC supplementation leads to an increase in GSH levels in heart, liver, and renal tissues, indicating strong protective potential.

**Figure 6:**
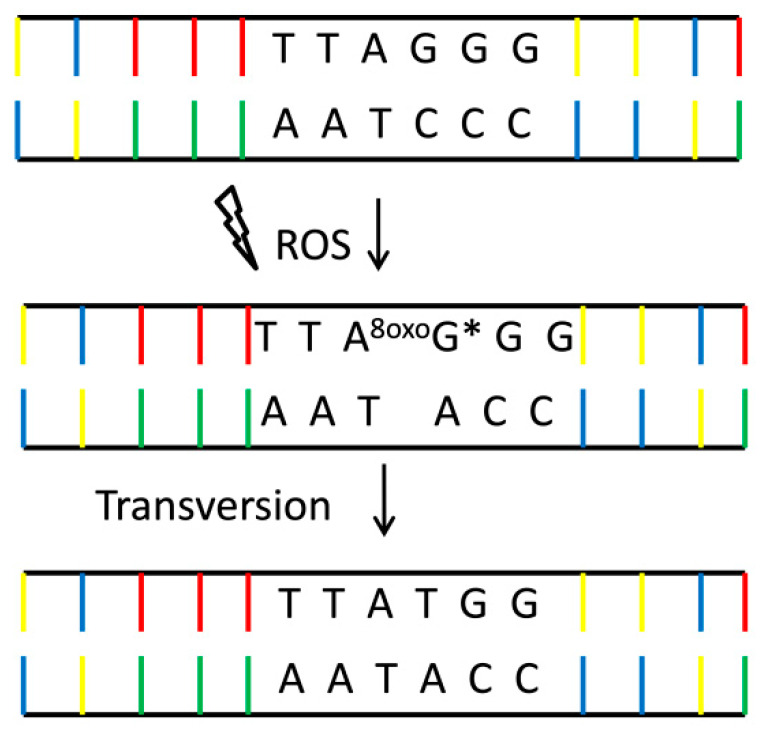
Mutagenicity of 8-oxoGua. 8-oxoGua—one of the most common DNA lesions resulting from ROS modification of guanine and leading to mismatched pairing with adenine, resulting in G to T and C to A substitutions in the genome. *~10,000 8-oxoGua residues per single nucleus of a human cell [87].

Base modifications, strand breaks or DNA adducts are the most common damage generated by ROS. The damaging factor is primarily the hydroxyl radical (^●^OH). Guanine (G) is the base that is easily oxidized, which causes 8-oxo-2-deoxyguanine (8-oxoGua) to be the most abundant DNA damage [87]. It is a mutagenic damage, because 8-oxoGua mispairs with adenine (A), which in the next replication cycle may generate a G to T transversion mutation [88] (Figure 6). As mentioned above, emerging lesions are recognized, among others, by the p53 protein, they trigger the activation of the checkpoint, which leads to cell cycle arrest. It enables the activation of DNA repair genes or apoptosis, and is aimed at maintaining genomic integrity after DNA damage [89].

8-oxodGTP is reported to constitute 1–10% of the dGTP pool (comparing to unmodified ones) [90]. Further GC-rich sites, such as telomeres (TTAGGG) or transcription factor binding sites are particularly vulnerable to oxidative damage and their mutation may change the gene expression profile [91]. DNA damage is a crucial concept for cancer therapies. The purpose of chemo or radiotherapy in the treatment of cancer is primarily DNA damage. DNA repair capacity in cancer is decreased and this consequently leads to cell death. Recent studies adopt dietary agents in combination with drugs to enhance their therapeutic effect targeting DNA repair pathways [92,93,94].

Despite the concerns and doubts that vitamin C may have a pro-oxidative effect, most evidence indicates vitamin C as a DNA protector. In cells subjected to oxidative stress (5 mM H_2_O_2_ and 100 µM Cu^2+^ which created an environment conducive to oxidation of G by Fenton reaction), an increased frequency of G to T transversion was observed. However, cells that were previously ascorbate treatment (500 µM DHA) had a significantly lower level of damage compared to control. Moreover, the reduction of glutathione levels in cells did not abolish the antimutagenic effect of vitamin C. Despite the pro-oxidative properties of vitamin C known under these conditions, the authors postulate that the antioxidant potential is much stronger, which directly inhibits the formation of 8-oxodG (8-oxo-2-deoxyguanosine) in an environment of high DHA levels in vitro [88]. In turn, studies on healthy people supplemented with vitamin C (500 mg per day) show initially an increase in 8-oxodG levels in plasma and urine of subjects [95]. The authors suggest that vitamin C affects DNA repair mechanisms through a redox change. In a subsequent paper, was show upregulation of AP-1 (Activator protein 1) transcription factor after administration of ascorbate at a dose of 500 mg/d. The activity of the AP-1 depending on the redox status, modulates the NER repair process. The authors claim that ascorbate stimulates 8-oxodG reduction by redox shift, activating AP-1, which induction the NER repair system [96]. The hypothesis that vitamin C stimulates DNA repair by temporarily inducing oxidative stress was supported by gene expression analysis. A change in expression of several dozen genes was observed after CCRF-HSB-2 cell exposure to 150 µmol/L vitamin C or ascorbic acid 2-phosphate (AA2P) and incubated up to 24 h. GdC (deoxycytidine glyoxal) levels in culture supernatant are increased after vitamin C treatment which indicates enhanced repair processes—NER removes adducts from DNA. According to genomics analysis AA2P has a higher potential than AA. In the case of AA2P genes involved in the cell cycle, stress response, and DNA repair are induced which does not occur for ascorbate. Expression of following genes relevant to DNA repair is upregulated: *XRCC5* (3.05-fold), *ERCC* (2.49-fold), *RAD18* homologue (2.27-fold), and *RAD50* homologue (2.07-fold). However, the effect is short-lived leading to a positive adaptive response [97]. Other gene expression profiling studies on primary human dermal fibroblasts (GM5659) show the effect of AA and AA2P on DNA repair [98]. The cells were incubated with 100 µM of AA2P, or AA for 5 days (with daily repletion). The expression profiling showed modulation of 294 genes, including those involved in replication, cell cycle regulation, and DNA repair. Expression of Flap structure-specific endonuclease 1 (*FEN1*) and replication factor C (*RFC*) taking part in long-patch BER is enhanced by 2-fold and 2.1-fold, respectively; expression level of one of the glycosylases initiating BER, Nei endonuclease VIII-like 3 (*NEIL3*) increased 2.9-fold; *H2AX,* and *RAD51* which are essential for double strand breaks (DSBs) repair increased 2.1-fold and 2.5-fold, respectively. Induction of this set of genes by ascorbic acid 2-phosphate may help the cell to repair and neutralize the negative effects of oxidative lesions that arise during replication.

The upregulation of the hOGG1 (8-oxoguanine glycosylase) involved in BER repair was observed after vitamin C supplementation (300 mg) patients undergoing chronic hemodialysis, as well as a decrease in the amount of 8-hydroxy-2-deoxyguanosine (8-OHdG) in peripheral blood lymphocytes [99]. Furthermore, other data indicate no effect of vitamin C on hOGG1 expression. The authors consider that supplementation with antioxidants may be of particular importance for people with nutritional deficiencies because no significant effect of supplementation was observed in a well-nourished subject [100]. Very interesting observations concern skin fibroblasts obtained from patients with burns. Cells were used to study the effect of AA (250 µM) on 84 genes associated with oxidative stress [101]. Among 10 significantly upregulated genes Ring Finger Protein 7 (*RNF7*) was identified (3.38-fold increase in expression). RNF7 acts as a redox sensor and is involved in protein degradation and cell cycle progression. More interestingly, it is recently described as one of the proteins interacting with proliferating cell nuclear antigen (PCNA) [102]. PCNA interacts with numerous repair proteins: FEN1, APE1, uracil DNA glycosylase (UNG2), 3-methyladenine-DNA glycosylase (MPG), and polymerase ε (Polε) from BER; XPA and XPG from NER, and polymerase δ (Polδ), MutS homolog 3 and 6 (MSH3, MSH6) from MMR. Therefore, it may be assumed that the upregulation of *RNF7* after vitamin C supplementation may indicate to some extent correlation between ascorbate and DNA repair action enhancement.

It is known that smokers have significantly reduced levels of antioxidants in the body [103]. Cigarettes are a source of several thousand different chemical compounds, of which the vast majority can damage DNA, proteins, and other cellular structures directly or by generating massive free radicals and thus extremely increases the level of oxidative stress [104,105,106]. Moller et al. have shown that supplementation with vitamin C through antioxidant-rich vegetables and fruit intake (250 g/day of broccoli, containing 146 mg of vitamin C) can be particularly beneficial for smokers. The decrease in the level of oxidative DNA damage by reducing the amount of ENDOIII (endonuclease III) and FPG (formamidopyrimidine DNA glycosylase) sensitive site was observed after 10 days supplementation [107]. While the oxidative DNA damage level dropped, the repair enzymes (hOOG1, nucleoside diphosphate linked moiety X-type motif (NUDT1), and heme oxygenase 1 (HMOX1)) expression was not significantly affected by the diet. Their further study showed that the four-week intake of vitamin C (slow-release) resulted in a significant increase of DNA repair incision efficiency [100]. These results argue with previous observations when the effect of antioxidants, including vitamin C, on the level of DNA damage in smokers has been studied. However, the effect of VC was not significant, which was explained by a failure to reach baseline vitamin C levels for non-smokers [108].

Other studies show that vitamin C is a natural factor that protects against environmental pollution by showing anti-clastogenic effects. Higher levels of vitamin C in the plasma can compensate for the effects of air pollution by lowering the frequency of chromosomal aberrations. Genes coding enzymes involved in the repair of 8-oxoGua in BER (*hOGG1*, apurinic/apyrimidinic endodeoxyribonuclease 1 (*APEX1*), X-ray repair cross-complementation group 1 (*XRCC1*)) and NHEJ (DNA ligase 4 (*LIG4*), *XRCC4, XRCC5, XRCC6*) systems are selected. The urinary levels of 8-oxodG are lower in patients with higher plasma vitamin C levels. The authors speculate that higher levels of VC caused a significant increase in the expression of *XRCC1* in some subjects and subsequent increase in BER efficiency (which resulted in the lower oxidized base level in urine) [109]. XRCC1 is a scaffold protein that coordinates most of the stages of the short patch BER pathway. The authors suggest that the interaction of XRCC1 with OGG1 stimulates DNA glycosylase activity resulting in higher repair efficiency.

Ionization, and excitation of molecules in the living matter are the first link in the chain of transformations leading to the biological effect of ionizing radiation. Ionizing radiation is capable of directly damaging a biomolecule by ionizing it, or breaking its bonds. Ionizing radiation generates free radicals directly as a result of the ionization of, e.g., water. They then trigger a chain of secondary biological reactions and generate subsequent generations of ROS.

The radioprotective properties of vitamin C are widely commented in the literature. To examine the radioprotective potential of peripheral blood lymphocytes (PBL) after gamma irradiation, they were incubated with vitamin C (1 µg/mL) for 48 h. Vitamin C post-treatment has been observed, reduces the frequency of chromosomal aberrations by about 30% and significantly reduces the number of DNA breaks, which according to the authors indicates the impact on DNA repair processes [110]. Similar observations apply to lucid skin fibroblasts, which irradiated with gamma rays from ^137^Cs (6 Gy). Both pre-treatment and post-treatment AA-2G (2-glucopyranoside ascorbic acid) fibroblasts show radioresistance [111]. AA-2G is a stable form of ascorbic acid used to create cosmetics [112]. AA-2G pre-treated cells are more resistant to gamma radiation (6 Gy), and the effect of post-treatment was to reduce DNA damage (in this case DSBs). The strongest protective effect was noted for cells incubated with AA-2G both before and after irradiation [113]. Radioprotective properties of vitamin C have been reported for ultraviolet (UVB) irradiated fibroblasts. Vitamin C (100 and 500 mM) protects the epidermal surface both by stimulating collagen synthesis as well as inhibiting oxidative stress, apoptosis, and reducing the frequency of DNA damage [114]. More recent data confirm AA and AA-2G reduce the amount of DNA damage, and chromosomal aberrations generated by gamma rays and UVB (Maeda 2020). Promising results relate to research on patients. The use of antioxidants (2 g ascorbate, 1.2 g N-acetylcysteine (NAC), 600 mg lipoic acid, and 30 mg beta carotene) 15 min before scintigraphic diagnosis resulted in a significant decrease in the amount of DNA damage in peripheral blood lymphocytes. The authors postulate that supplementation with antioxidants may protect people particularly exposed to radiation, i.e., patients regularly subjected to scintigraphy tests or medical staff [115].

Different conclusions were presented by researchers studying the effect of vitamin C on lymphocytes of healthy persons and with endometrial cancer. The therapeutic concentration of vitamin C (20–80 µg/mL) sensitized lymphocytes to X-ray radiation (0.3 Gy) and showed dose-dependent co-mutagenicity. The authors conclude that VC at low doses of radiation may constitute a carcinogenic threat [116].

## 6. Conclusions

The current review shows that, in addition to the typical antioxidant activity, vitamin C can be beneficial as a pro-oxidative factor. Despite the observed elevated levels of markers of DNA damage, 8-oxodG in plasma, or urine as a result of VC supplementation, it is believed that vitamin C promotes the removal of 8-oxodG from DNA and the pool of nucleotides, by upregulation of repair enzymes due to pro-oxidative properties. By activating the AP-1 described earlier, it stimulates DNA repair processes, e.g., NER. Vitamin C, by generating H_2_O_2_ and the ^•^OH radical, can affect the viability of cancer cells sensitive to oxidative damage to DNA, membranes, or mitochondria. Last but not least, it has been shown that it can affect cell survival by stabilizing p53—the main controller of cell proliferation and apoptosis. Vitamin C has also been recognized for protection against radiation-induced cell damage.

Vitamin C and its effect on DNA damage and repair in living organisms seem to be dependent on many factors. Studies show that the baseline level of ascorbate in patients, and their general health condition may determine whether or not supplementation (including fruit and vegetable consumption) will have a beneficial influence. For poorly nourished patients increase in vitamin C levels caused beneficial effects on DNA integrity [100]. Many studies provide ambiguous information about the effects of vitamin C on human cells and the body. As for in vitro and genome analysis studies results indicate that VC acts variously in different cell lines and conditions. This makes more difficult for researchers to come to the final conclusion whether or not vitamin C, and the positive effect and potential of enhancing DNA repair processes.

The concentration of vitamin C in plasma depends on such factors as: body weight, smoking, dietary supplements, lifestyle, food processing techniques, and health status. Importantly, an inverse correlation of plasma ascorbate concentration with age is observed (with age, ascorbate levels in lymphocytes and plasma decrease by approximately 20%) [117,118]. The recommended dietary allowance (RDA) for vitamin C of 75 mg for women and 90 mg for men is, based on the vitamin’s role as an antioxidant as well as protection from deficiency [119]. The vitamin C plasma concentration (after oral intake) is controlled at 70–85 µmol/L for amount as much as 300 mg daily that can be obtained from food [6]. In healthy people, amounts greater than the RDA do not appear to be helpful. Vitamin C nutritive may be more important for people with certain diseases or conditions. The demand for vitamin C increases during lactation, with heavy physical exertion, in smokers, alcoholics, the elderly, and those suffering from hypertension and diabetes. These people daily should take more than 120 mg of vitamin C. There are recommendations to consume 3 g of vitamin C per day, but side effects may occur: nausea, vomiting, and diarrhea. As mentioned before excess vitamin C can cause excessive iron absorption, and impair the absorption of vitamin B12, and copper [120]. Despite the high intakes of the vitamin that are generally well tolerated a tolerable upper level was set at 2 g based on the gastrointestinal upset that sometimes accompanies excessive intakes [121].

Despite the controversy, whether vitamin C can be a panacea for many diseases, it seems that it can support standard treatment regimens. This, however, requires firm and unequivocal support of the results of clinical trials and tests. Vitamin C, although present in the consciousness and diet of people for decades, should not be underestimated or overestimated. Ascorbate is an active compound that affects many intracellular processes including epigenetic regulation of gene expression, intracellular signaling [122], and of course switching redox status. An unequivocal explanation is needed as to whether ascorbate can affect the instability of human somatic cells or is definitely a protective factor.

## Figures and Tables

**Table 1:** Approximate ascorbate concentration in human tissue and cells according to [14,72].

Tissue/Cells	Ascorbate (mM)	Ref.
Plasma, healthy	0.04–0.08	[14,72]
Lymphocyte	3.8–4	[14,72]
Platelet	2.7–3.7	[14,72]
Red blood cell	0.045	[14,72]
Brain	0.8–0.9	[14]
Cerebral spinal fluid	0.15–0.25	[14,72]
Pituitary gland	2.3–2.8	[14]
Neuron	10	[72]
Glial cells	1	[72]
Leans	1.4–3.4	[14,72]
Adrenal	2	[72]
Kidney	0.3–0.9	[14]
Lung	0.4	[14]
